# COVID-19 and flu: conserved or specific immune signature?

**DOI:** 10.1038/s41423-020-00595-3

**Published:** 2021-01-11

**Authors:** Christophe Paget, François Trottein

**Affiliations:** 1 Centre d’Etude des Pathologies Respiratoires, INSERM U1100, Université de Tours, Faculté de Médecine de Tours, 37000 Tours, France; 2 Centre d’Infection et d’Immunité de Lille, INSERM U1019, CNRS UMR 9017, Université de Lille, CHU Lille, UMS 2014 - PLBS, U1019, Institut Pasteur de Lille, 59000 Lille, France

**Keywords:** Predictive markers, Diagnostic markers

Respiratory viruses are a major cause of infectious disease and mortality worldwide. Among respiratory viruses, emerging and well-known coronaviruses and influenza viruses cause seasonal epidemics and pandemics and rank as very important pathogens. The clinical signs of viral respiratory infections range from asymptomatic forms to life-threatening acute respiratory distress syndrome (ARDS, associated with a high mortality rate). Another reported cause of mortality is multiple organ dysfunction. The ongoing pandemic of coronavirus disease 19 (COVID-19, caused by severe acute respiratory syndrome coronavirus 2 (SARS-CoV-2)) is a serious public health problem. SARS-CoV-2 infection induces to a clinical signature distinct from that associated with other types of pneumonia, and SARS-CoV-2 differs from influenza viruses with regard to target cells and replicative properties. The morbidity and mortality rates are higher for COVID-19 than for seasonal influenza.

When considering community-acquired viral pneumonia caused by different pathogens, it is essential to compare the respective host immune signatures. This comparison can highlight conserved or specific immune pathways involved in the pathogenesis. In a recent issue of *Immunity*, Zhu et al. described the dynamic immune landscape in patients with SARS-CoV-2 or influenza A virus (IAV) infections.^1^ Using single-cell RNA sequencing, the researchers compared transcriptomic changes during the course of infection in peripheral blood mononuclear cells (PBMCs). This approach enabled the researchers to report that the gene expression pattern in patients with COVID-19 differs from that in IAV-infected patients.

**Fig. 1:**
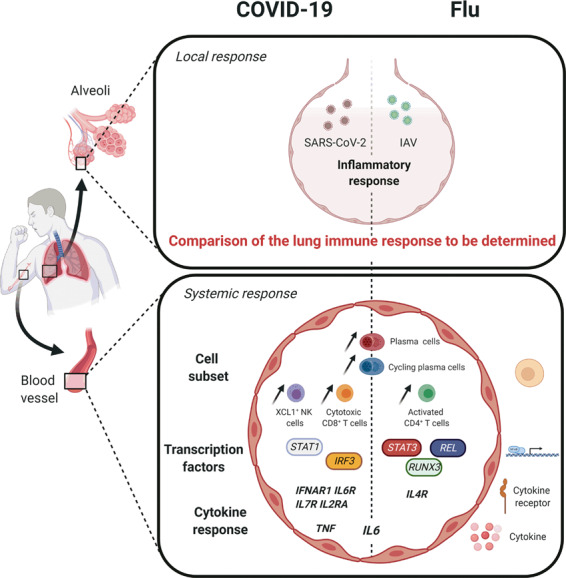
Local and systemic responses during COVID-19 and influenza infections. Conserved and specific immune pathways (cell subsets, transcription factors and cytokines) potentially involved in the pathogenesis were identified at the systemic level. Further work is needed to analyze specific and common immune traits in the lungs during COVID-19 and influenza infections

An average of 2000 unique transcriptomes for each patient and at each time point were generated. Unsupervised clustering of these transcriptomes identified 15 discrete cell populations. None of the cell populations analyzed presented signs of viral genetic material. Among all clusters, the proportion of some B cell subsets (plasma and cycling plasma cells) was greater in all patients with viral infection than in healthy controls, suggesting an active mechanism of producing protective antibodies (as exemplified by the upregulation of signature genes such as *PRDM1* and *IRF4* in the B cells). This indicates that on admission, patients have already started to mount a virus-specific immune response. Compared to IAV-infected patients, the counts of XCL1^+^ NK cells and CD8^+^ T cells were significantly increased in patients with COVID-19. Conversely, IAV-infected patients had an increased count of activated CD4^+^ T cells. Regarding the molecular components of the immune response, the expression of proinflammatory cytokines (*TNF*) and cytokine receptors (*IL6R*, *IL2RA,* and *IL7R*) was higher in multiple cell types in patients with COVID-19 than in IAV-infected patients. Of note, many receptors involved in the activation of cytokines were more strongly expressed in activated CD4^+^ T cells, indicating a particular role of this subset in the cytokine-driven inflammatory response reported in COVID-19 patients. The expression levels of hallmark genes in the type I IFN response (*STAT1*, *IRF3*, and *IFNAR1*) were also increased in the circulating immune cells of patients with COVID-19. This is of interest with regard to the controversial role of type I IFNs in the host response to SARS-CoV-2 infection.^2^ Conversely, IAV-infected patients had enhanced expression of transcription factors (such as *STAT3*, *REL,* and *RUNX3*) involved in the regulation of the inflammatory immune response when compared with the expression in patients with COVID-19. Taken as a whole, Zhu et al.’s results suggest that patients with COVID-19 and patients with influenza have both common and specific systemic immune traits (Fig. 1). Interestingly, a recent report also compared the immune responses in patients with COVID-19 and those with IAV infections.^3^ Lee et al. confirmed the intense type I IFN response in patients with severe COVID-19, reinforcing the hypothesis of a pivotal role played by type I IFN in exacerbating cytokine-mediated inflammatory responses in patients with severe COVID-19.

Although these data provide important insights and reveal differences in the immune landscapes in patients infected with SARS-CoV-2 and those infected with influenza viruses, they also raise new questions. The small number of patients in each group (5 and 8 patients with COVID-19 in Zhu et al.’s and Lee et al.’s studies, respectively) might accentuate individual differences. Single-cell transcriptomics has revealed the heterogeneity of peripheral immune activation in patients with COVID-19.^4^ To gain a detailed, robust view of the systemic immune responses, these data need to be confirmed in future prospective cohorts with larger patient groups. Since neutrophil dysfunction has emerged as a potential key event in COVID-19 pathogenesis,^5^ comparative analyses using total circulating leukocytes instead of PBMCs should be encouraged. Advanced integration of the transcriptomic data with the clinical presentation and management (e.g., the nature of supportive care and treatments) would also be useful. Longitudinal analyses of well-characterized clinical cohorts (in terms of age and comorbidities) will be essential. Complementary high-throughput technologies (such as Cellular Indexing of Transcriptomes and Epitopes by Sequencing, metabolomics, and proteomics) might also be very informative.^6^ Exploring the mechanisms that underlie the transition from respiratory virus-related pneumonia to ARDS and multiple organ dysfunction is critical for furthering our understanding of the pathogenesis of COVID-19 and flu. Therefore, studies of patients with various clinical courses and outcomes will be highly valuable. Since the inflammatory response in viral infection-induced ARDS (including COVID-19-driven ARDS) is compartmentalized within the lung,^7,8^ a comparative analysis of the immune landscape in the lung will be of great interest. It is noteworthy that single-cell omics of the upper respiratory tract^9^ and bronchoalveolar lavages^10^ has already revealed interesting clues. Finally, although scientists are usually conditioned to identify differences and to generate original data, highlighting common immune traits in distinct diseases might also be useful for advancing our understanding of the pathogenesis and developing therapeutic strategies. Moreover, immunologists should be encouraged to create a comprehensive and dynamic picture of the immune response during acute viral respiratory infections. This type of approach might yield valuable predictive markers of disease severity and outcomes and might pave the way for generic or more tailored treatment options.
